# Supplementary material for: Targeting PIKfyve-driven lipid metabolism in pancreatic cancer
Source: Nature. 2025 Apr 23;642(8068):776–84. doi: 10.1038/s41586-025-08917-z (PMC12176661; doi:10.1038/s41586-025-08917-z)
Supplement: Supplementary file 1 — This file contains Supplementary Fig. 1 (raw, uncropped western blot images) and Supplementary File 1 (the chemical synthesis of PIK5-33d). [file 41586_2025_8917_MOESM1_ESM.pdf]

nature portfolio

https://doi.org/10.1038/s41586-025-08917-z

Supplementary information

Targeting PIKfyve-driven lipid metabolism in pancreatic cancer

In the format provided by the authors and unedited

Fig 2a- 7940B

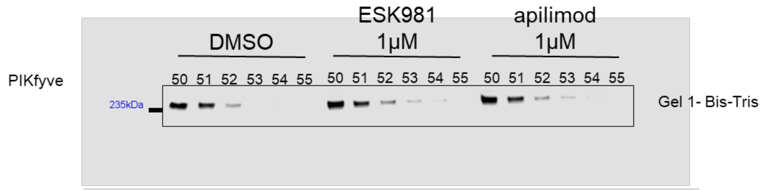

DMSO
ESK981
1μM
apilimod
1μM
PIKfyve
50 51 52 53 54 55 50 51 52 53 54 55 50 51 52 53 54 55
235kDa
Gel 1- Bis-Tris

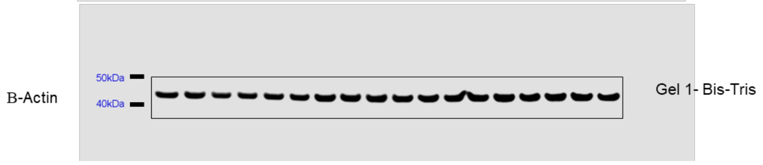

B-Actin
50kDa
40kDa
Gel 1- Bis-Tris

Fig. 4a- 7940B (trametinib, tram)

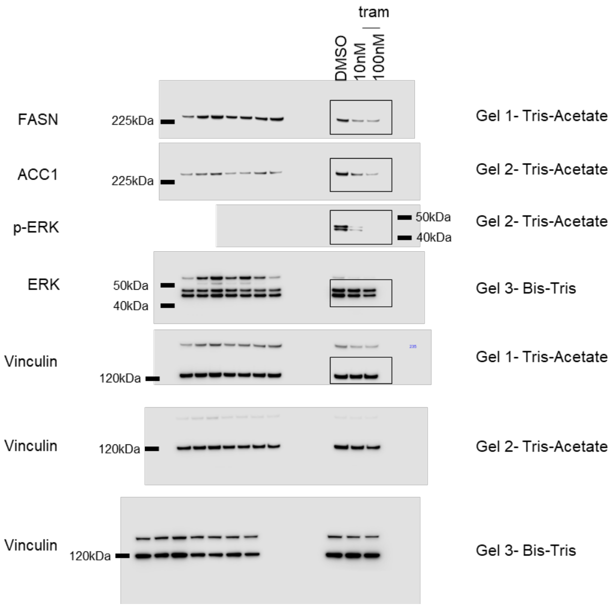

Fig. 4a- 7940B (MRTX1133, MRTX)

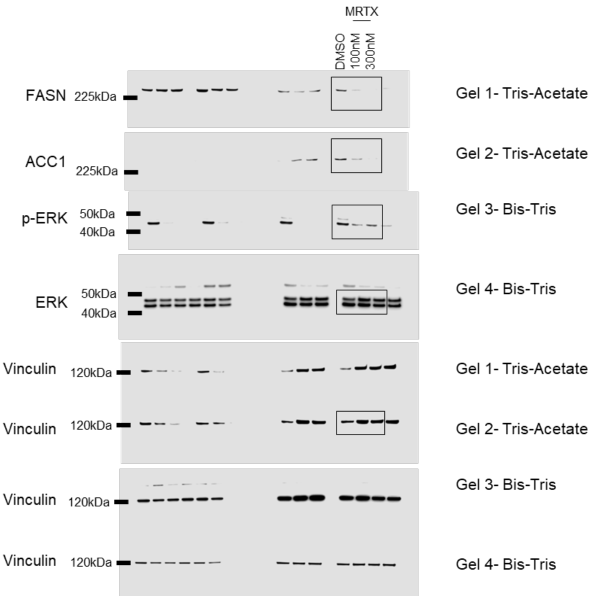

Fig. 4c- iKras 9805

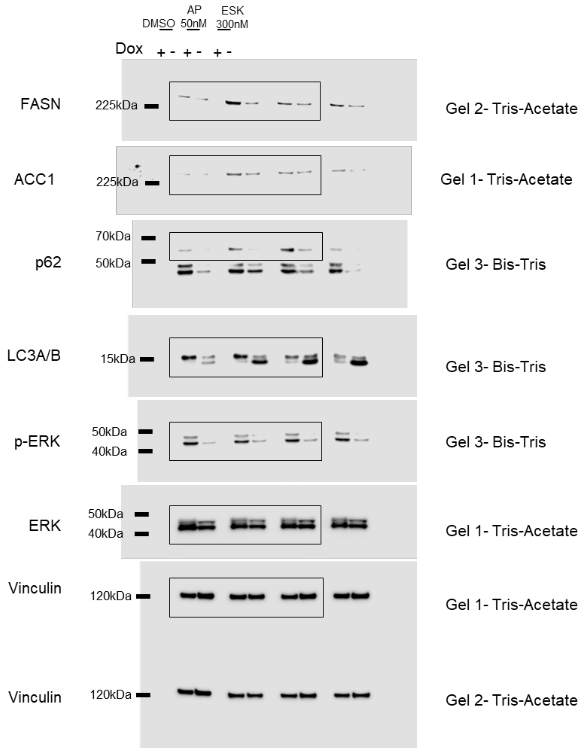

Extended Data Fig. 1h- murine pancreata

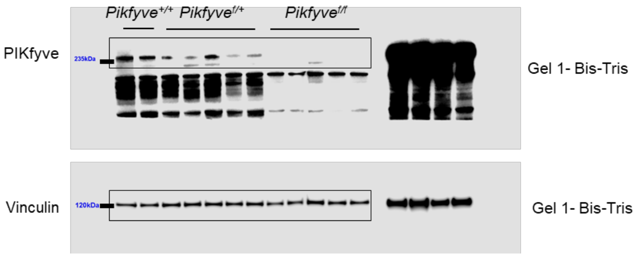

Extended Data Fig. 3s- UM2 tumors

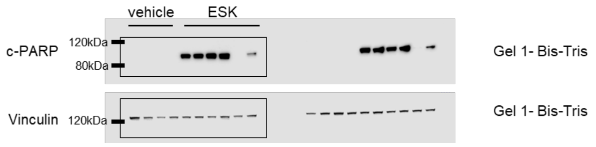

Extended Data Fig. 4b- MIA PaCa-2

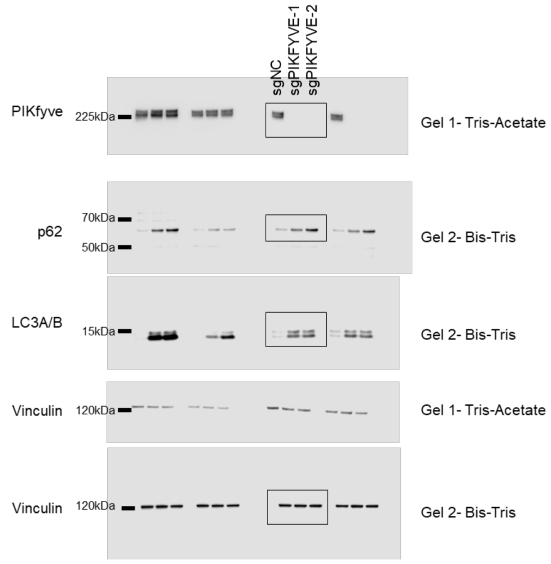

Extended Data Fig. 4b- PANC1- same samples as used in Extended Data Fig. 6g

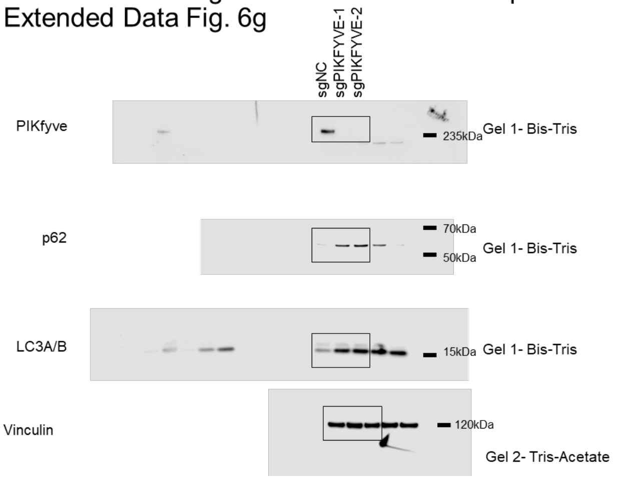

Extended Data Fig. 4c- 7940B

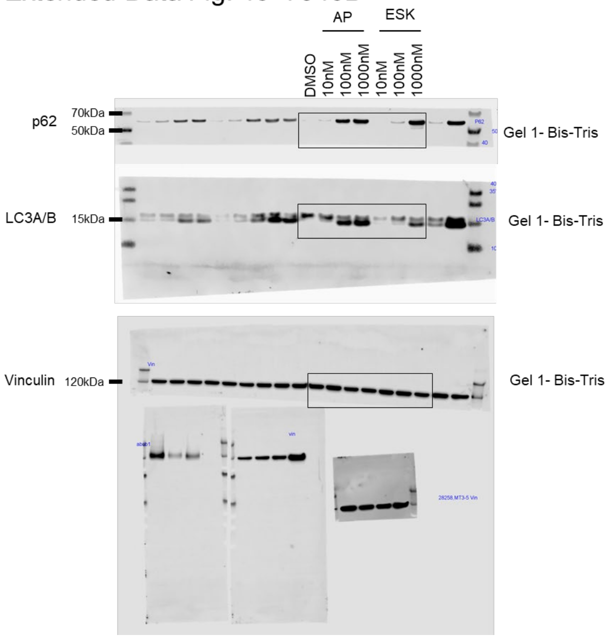

Extended Data Fig. 4c- Panc 04.03

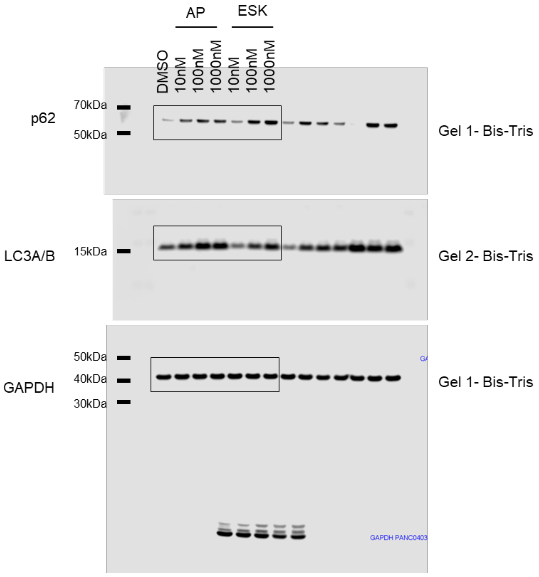

Extended Data Fig. 4d- UM2 primary (same samples as Extended Data Fig. 3s)

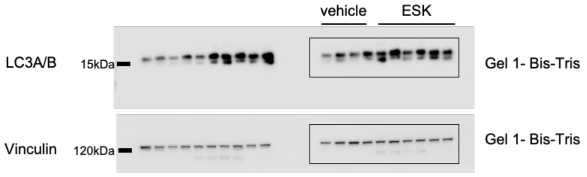

Extended Data Fig. 4h-7940B

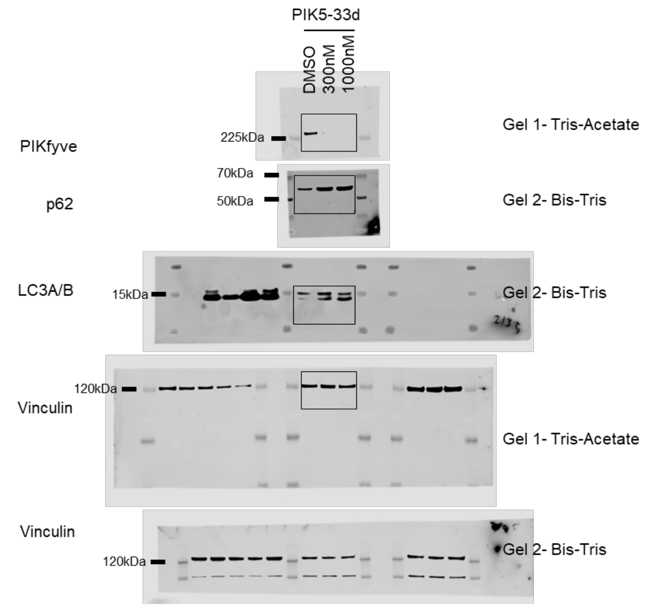

Extended Data Fig. 4h- MIA PaCa-2

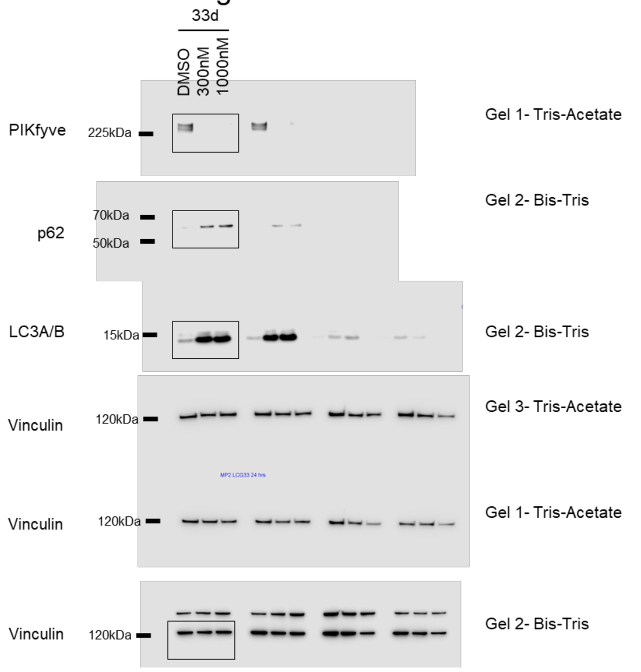

Extended Data Fig. 4t- 7940B

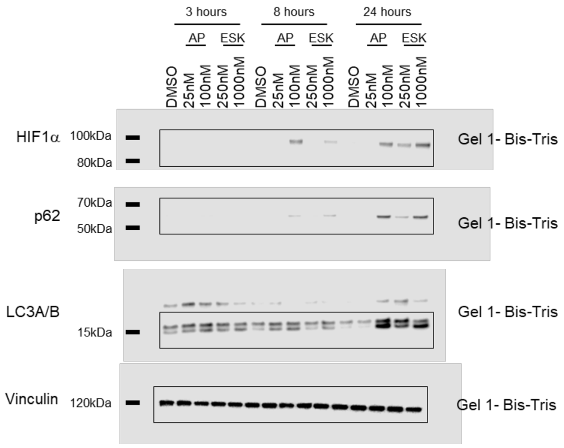

Extended Data Fig. 5e- MIA PaCa-2

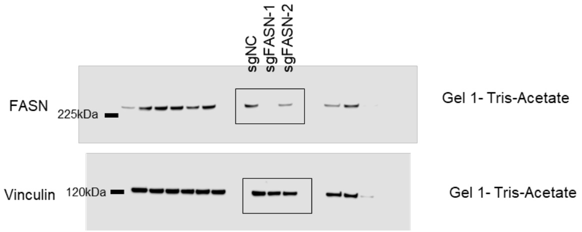

Extended Data Fig. 5h- PANC1, 7940B, and MIA PaCa-2

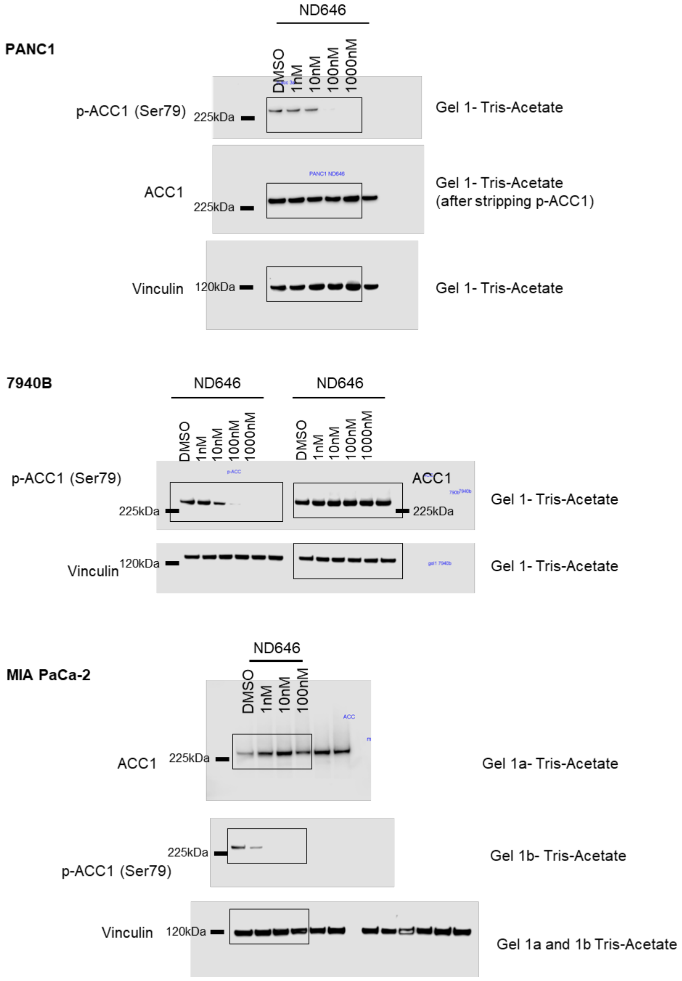

Extended Data Fig. 5m- PANC1 and MIA PaCa-2

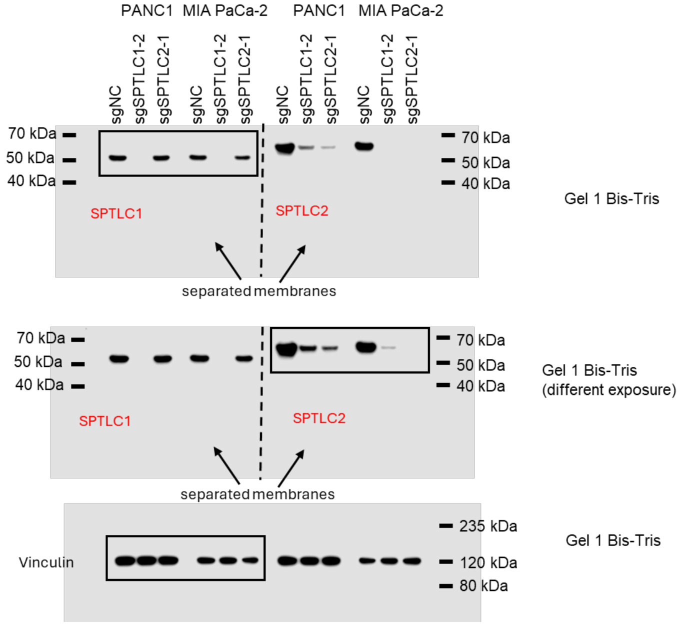

Extended Data Fig. 6e- MIA PaCa-2

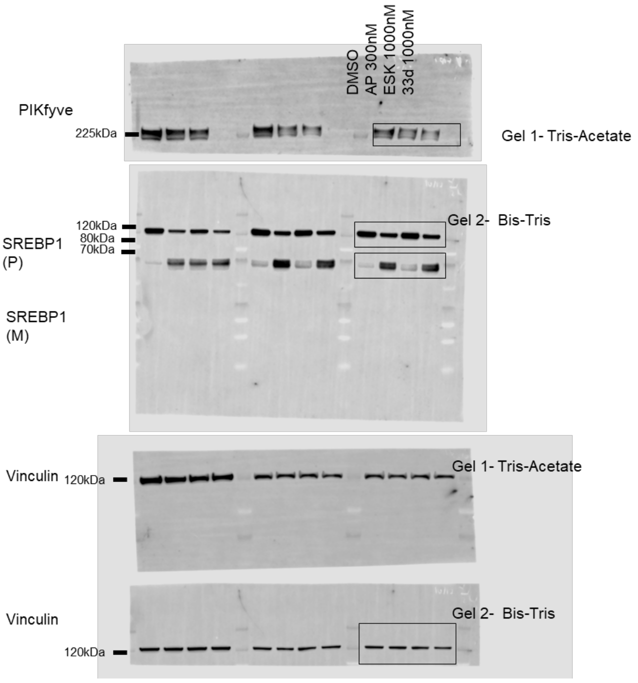

Extended Data Fig. 6e- PANC1

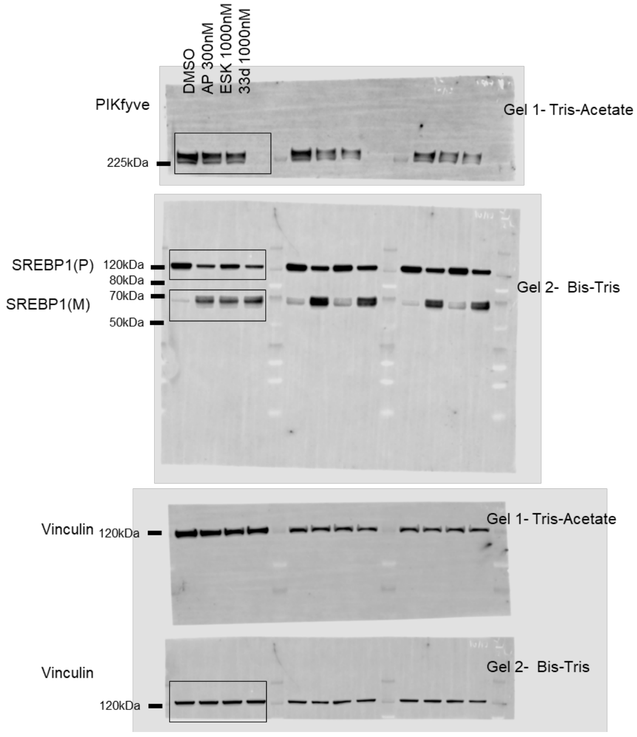

Extended Data Fig 6e- 7940B

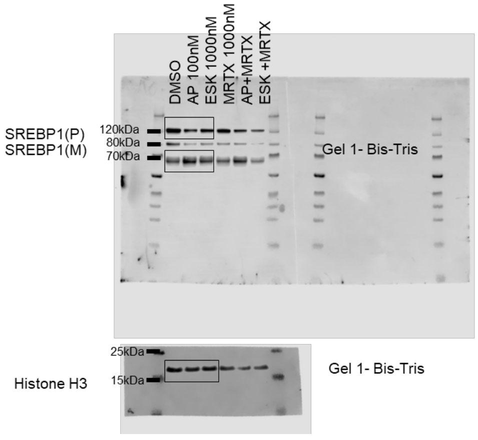

Extended Data Fig. 6g- PANC1 and MIA PaCa-2. The PANC-1 samples are from the same experiment as Extended Data Fig. 4b

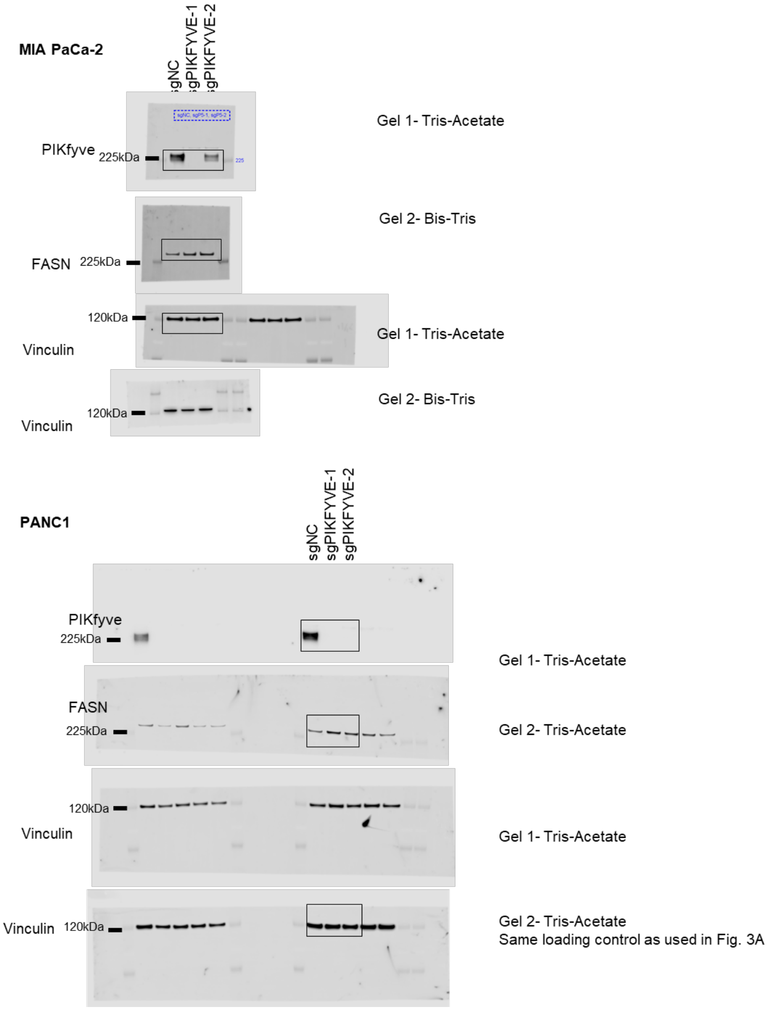

Extended Data Fig. 6i- MIA PaCa-2

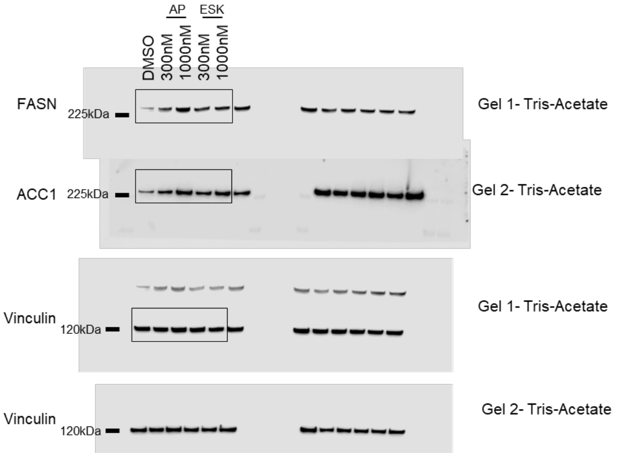

Extended Data Fig. 6i- PANC1

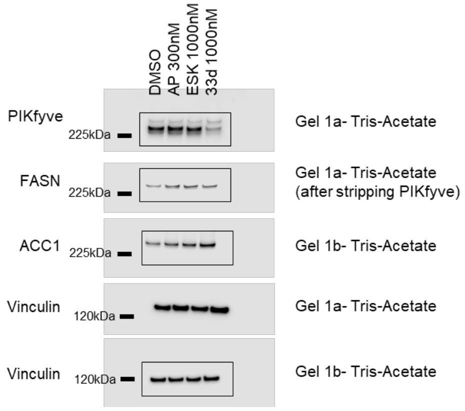

Extended Data Fig. 6j- 7940B (AP)

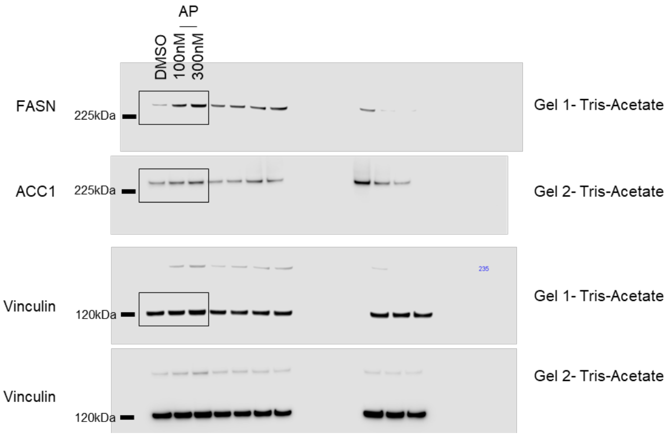

Extended Data Fig. 6j- 7940B (ESK)

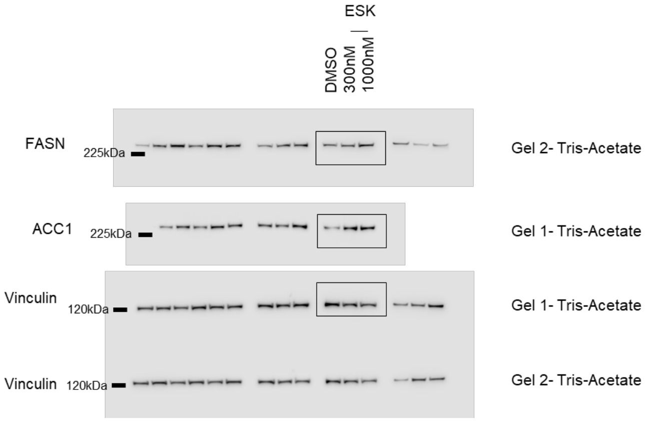

Extended Data Fig. 6j- 7940B (PIK5-33d)

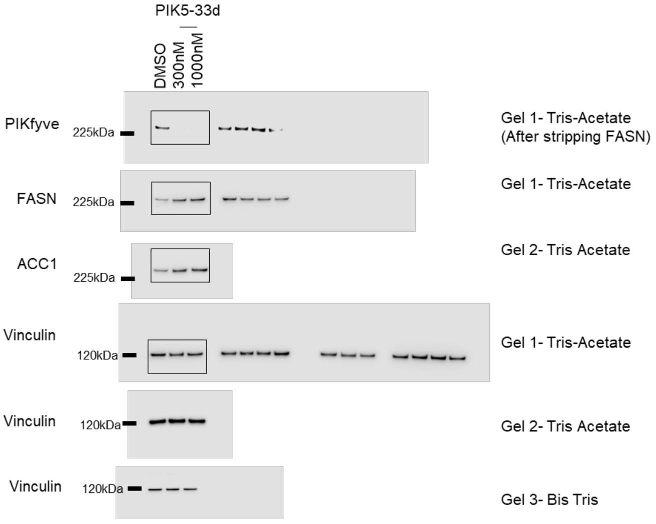

Extended Data Fig. 6k- 7940B, MIA PaCa-2, and PANC1

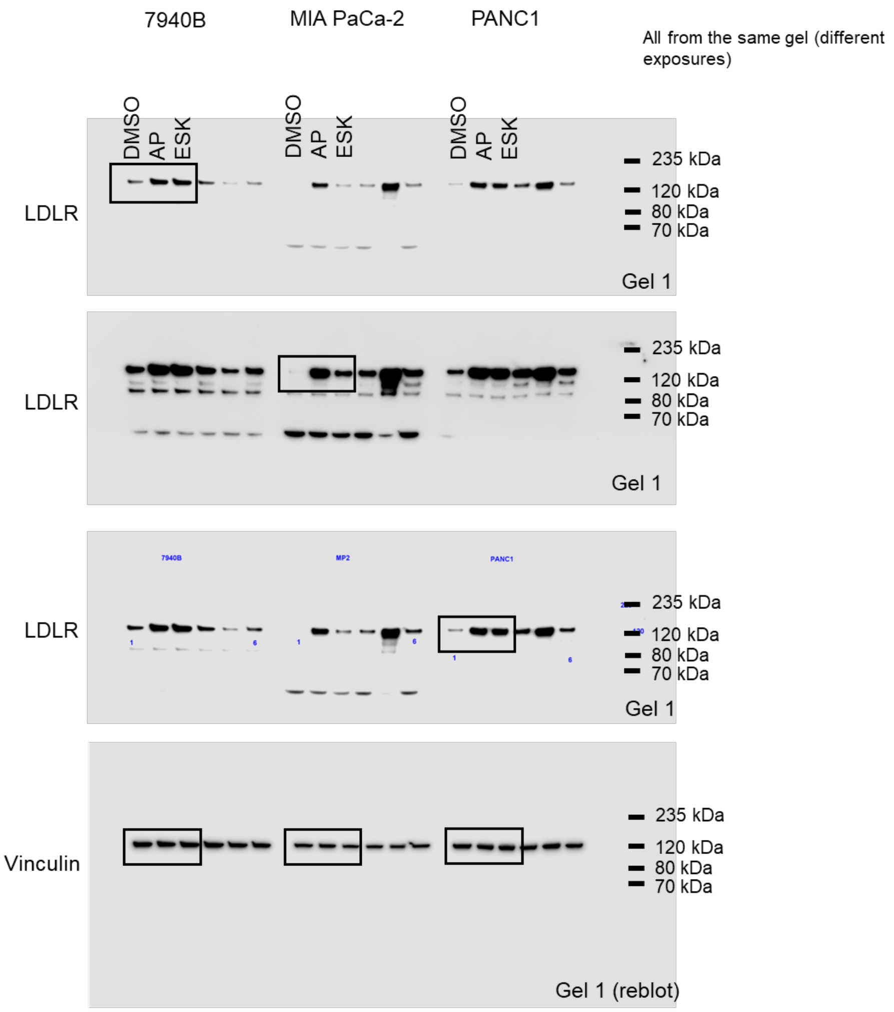

Extended Data Fig. 6m-7940B, PANC1, and MIA PaCa-2

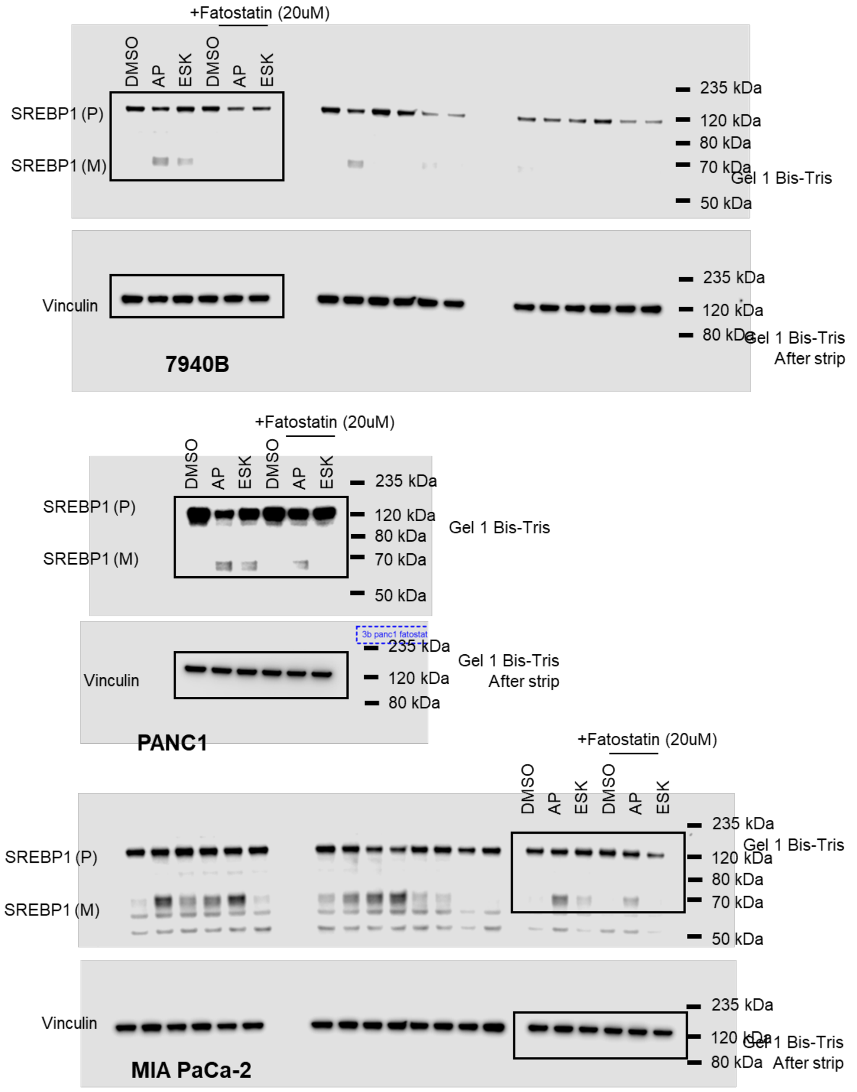

Extended Data Fig. 8a- 790B, PANC1, and MIA PaCa-2

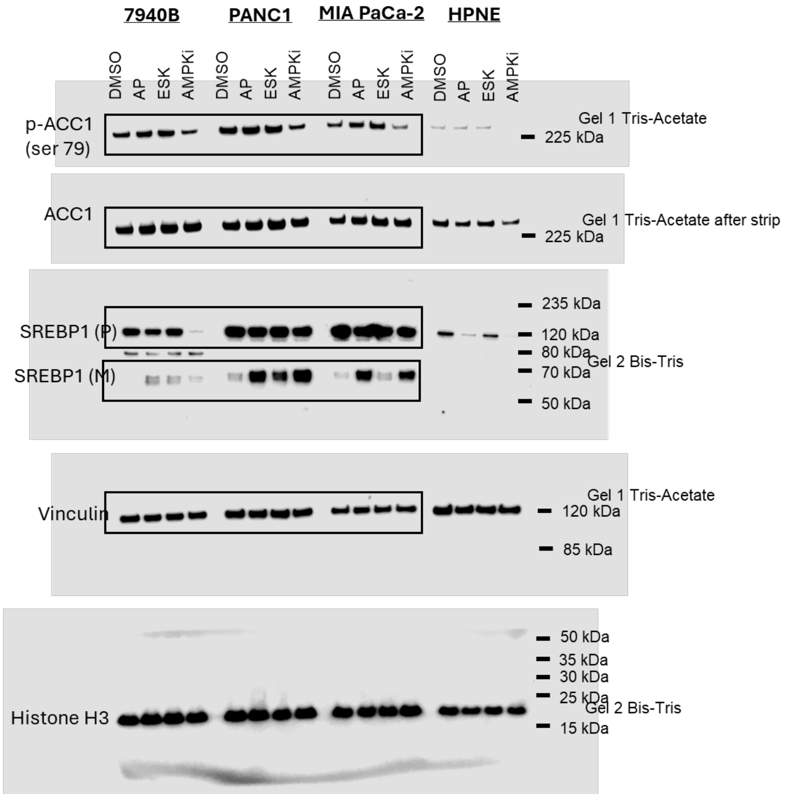

Extended Data Fig. 8b- MIA PaCa-2

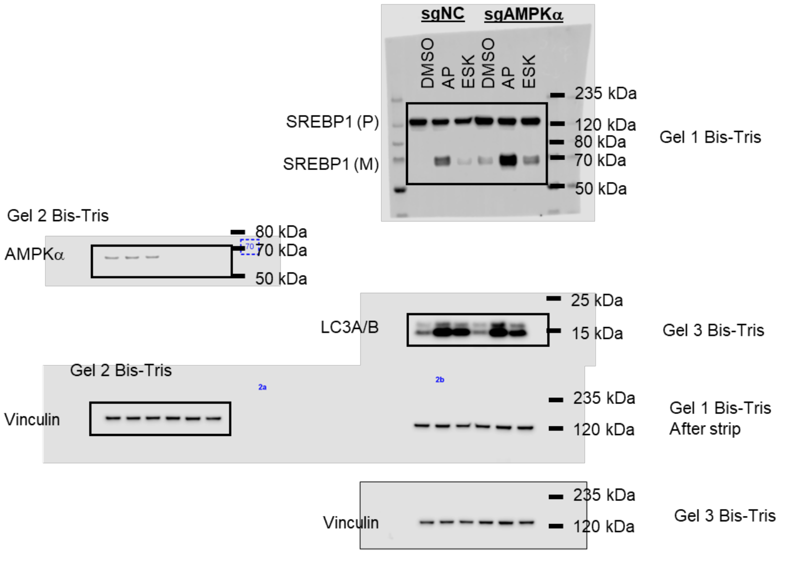

Extended Data Fig. 8e- PANC1

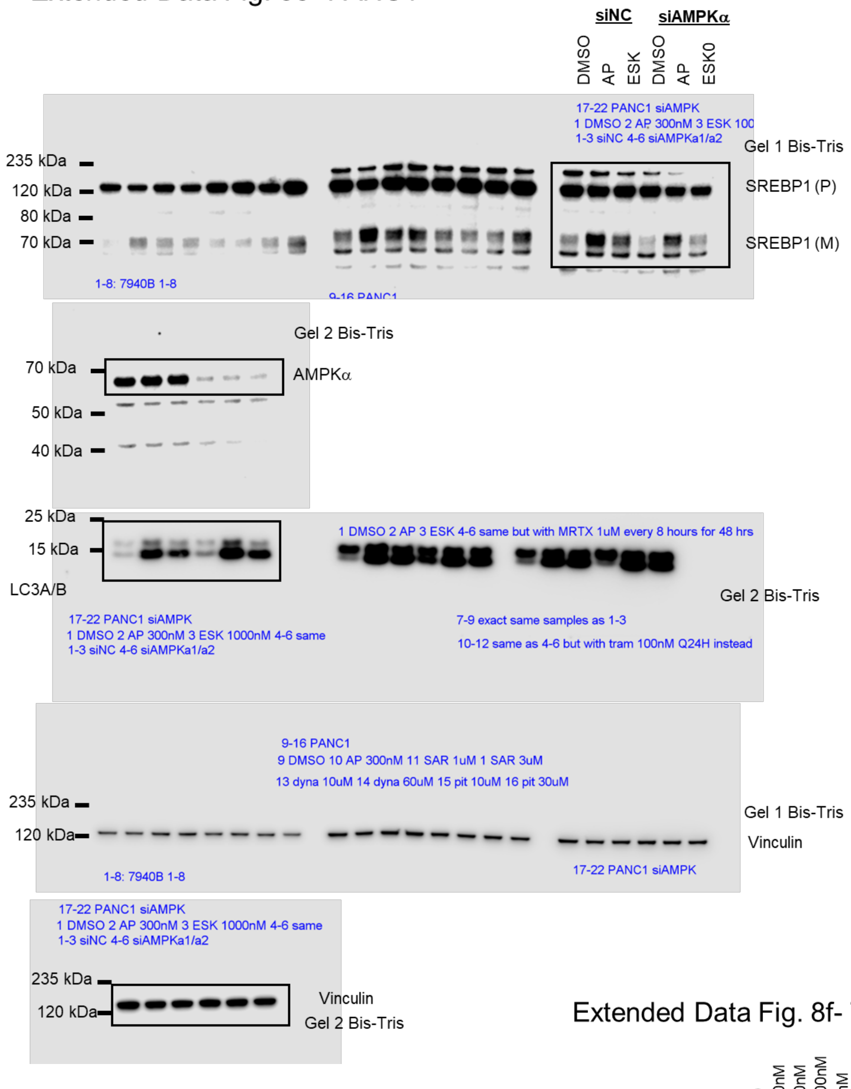

Extended Data Fig. 8f- 7940B

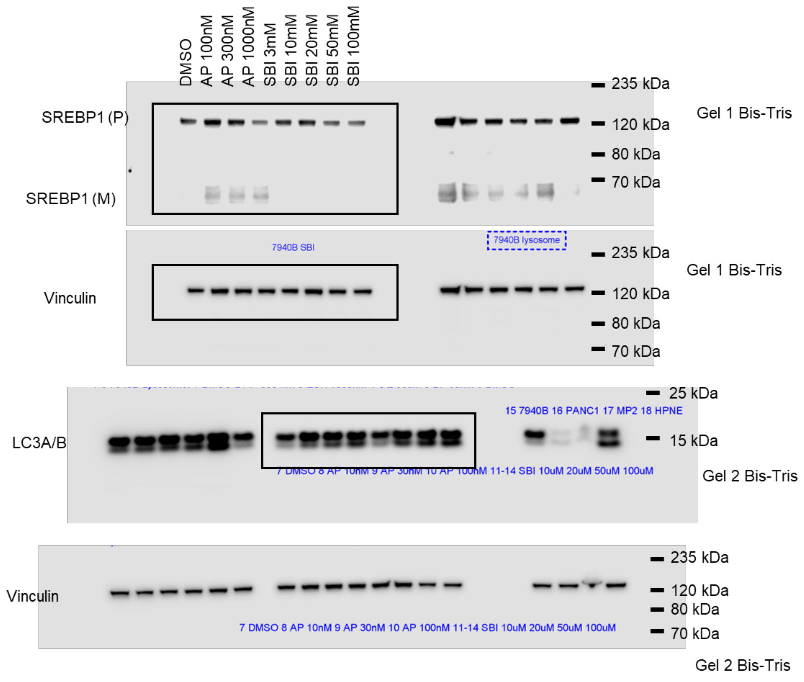

Extended Data Fig. 8g- MIA PaCa-2

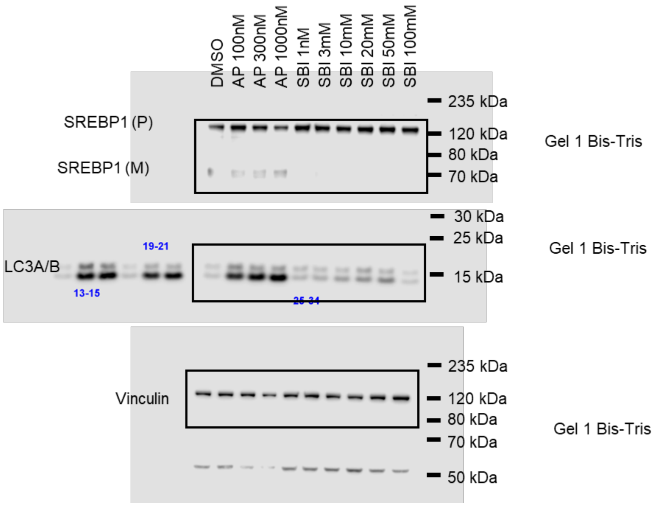

Extended Data Fig. 8h- PANC1

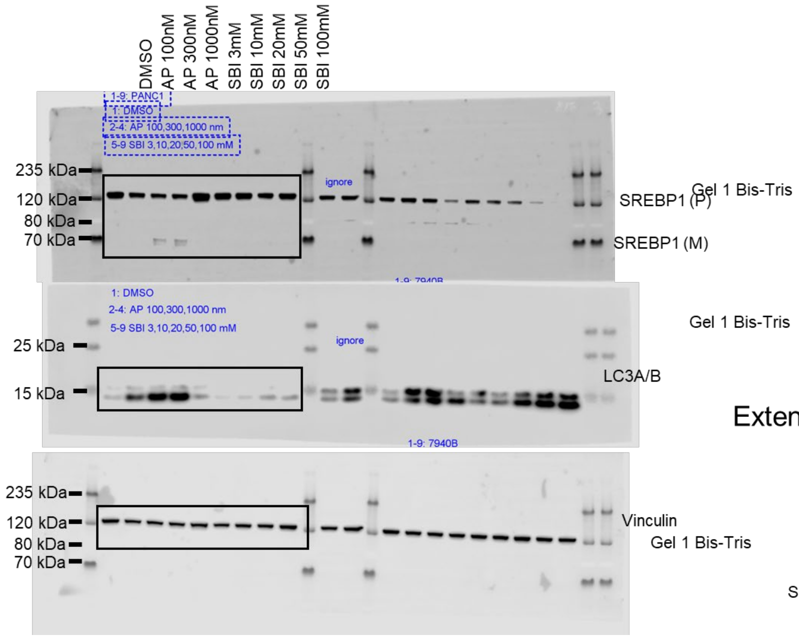

Extended Data Fig. 8i- KPC1361

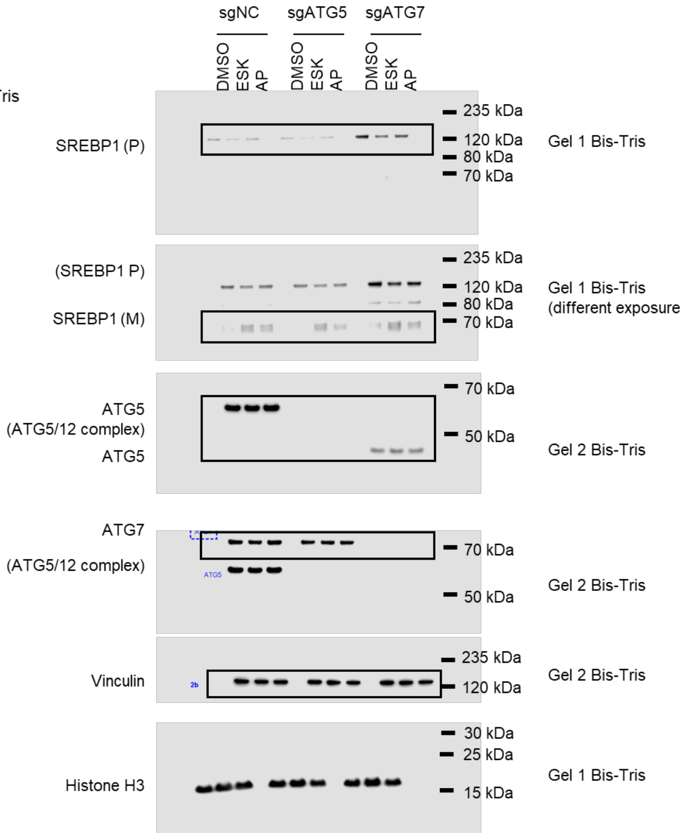

Extended Data Fig. 9a – 7940B

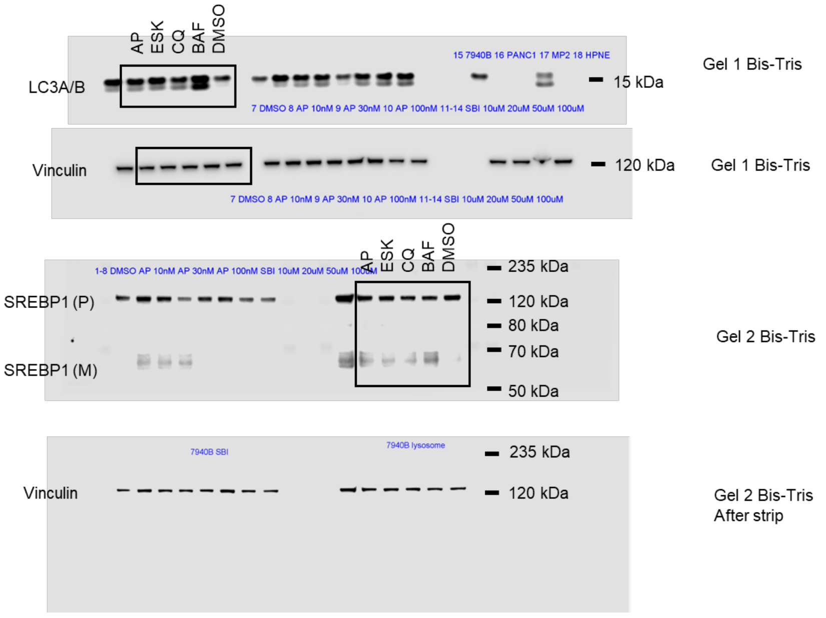

Extended Data Fig. 9a- PANC1

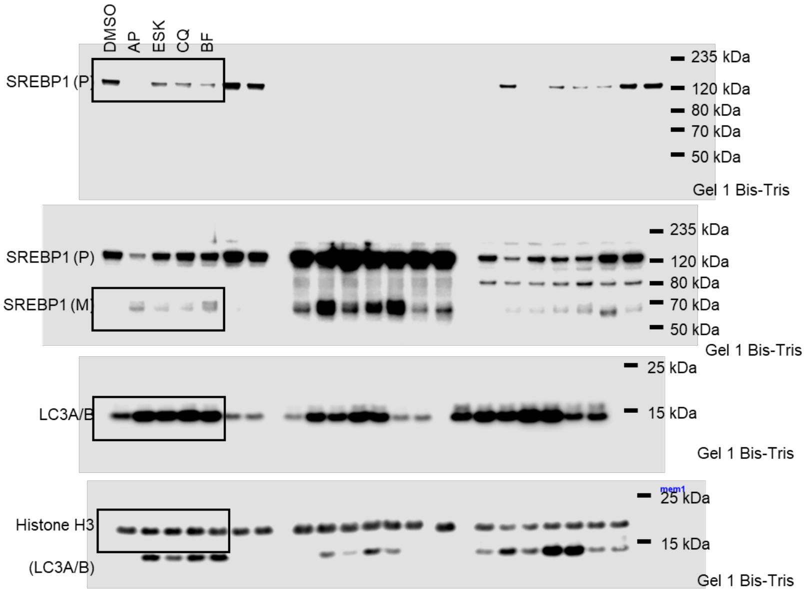

Extended Data Fig. 9a- MIA PaCa-2

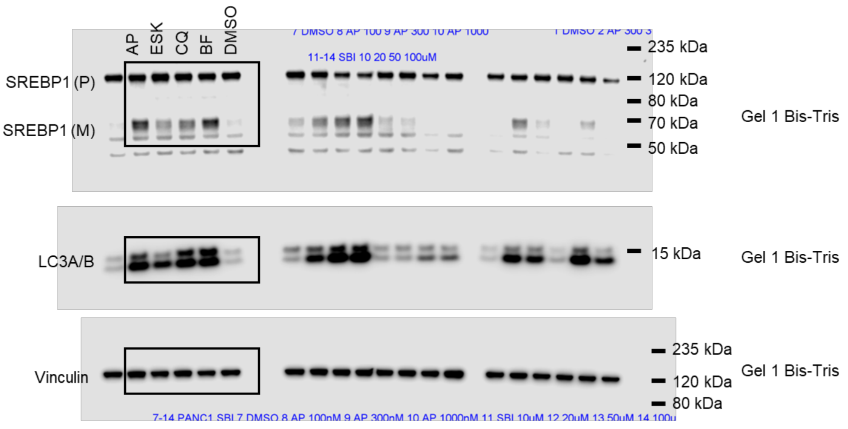

Extended Data Fig. 9a- HPNE

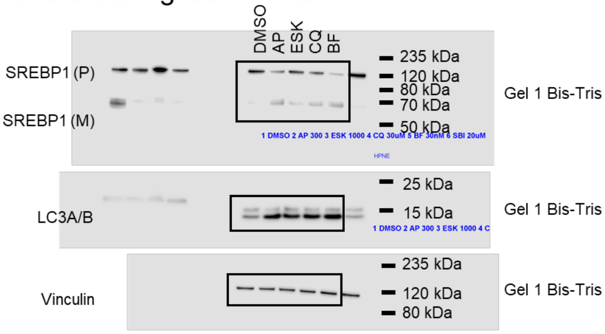

Extended Data Fig. 9c- MIA PaCa-2 and PANC1

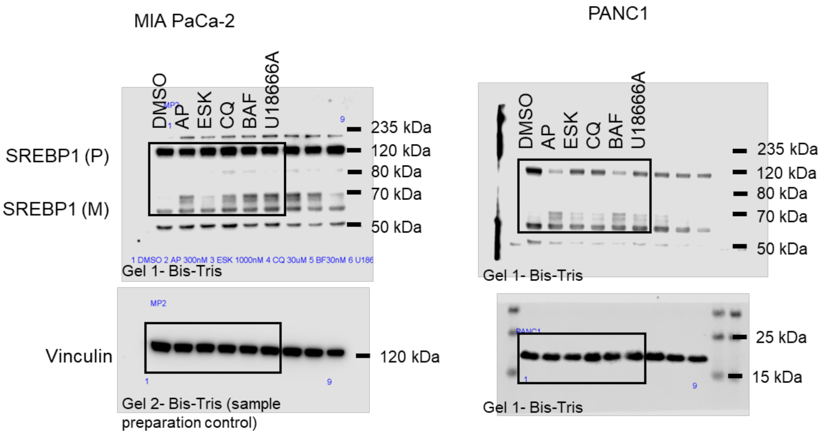

Extended Data Fig. 10a- PANC1 and MIA PaCa-2

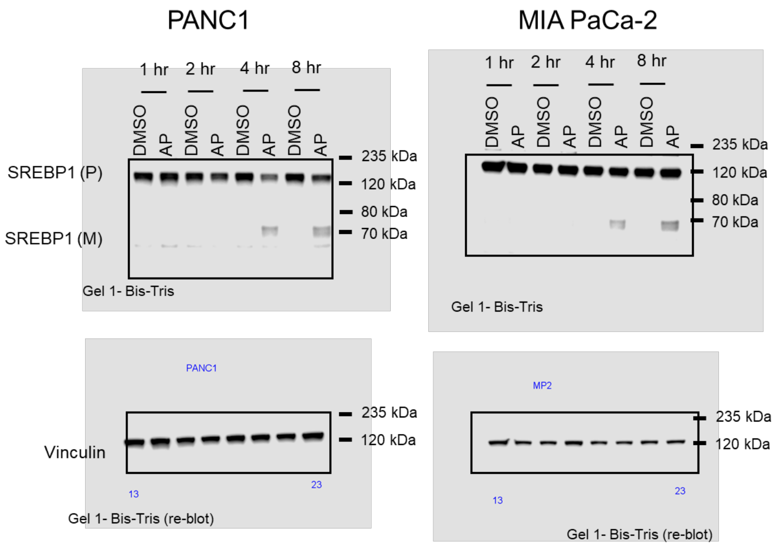

Extended Data Fig. 10e- MIA PaCa-2

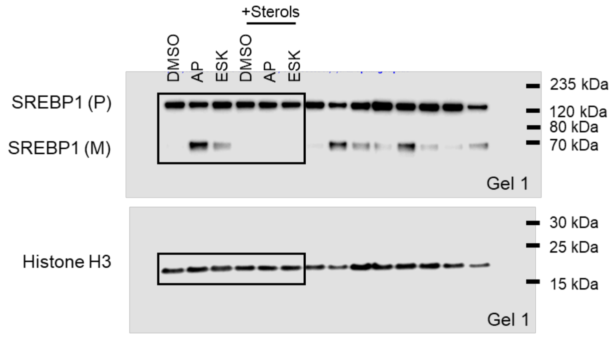

Extended Data Fig. 10e- PANC1 and 7940B

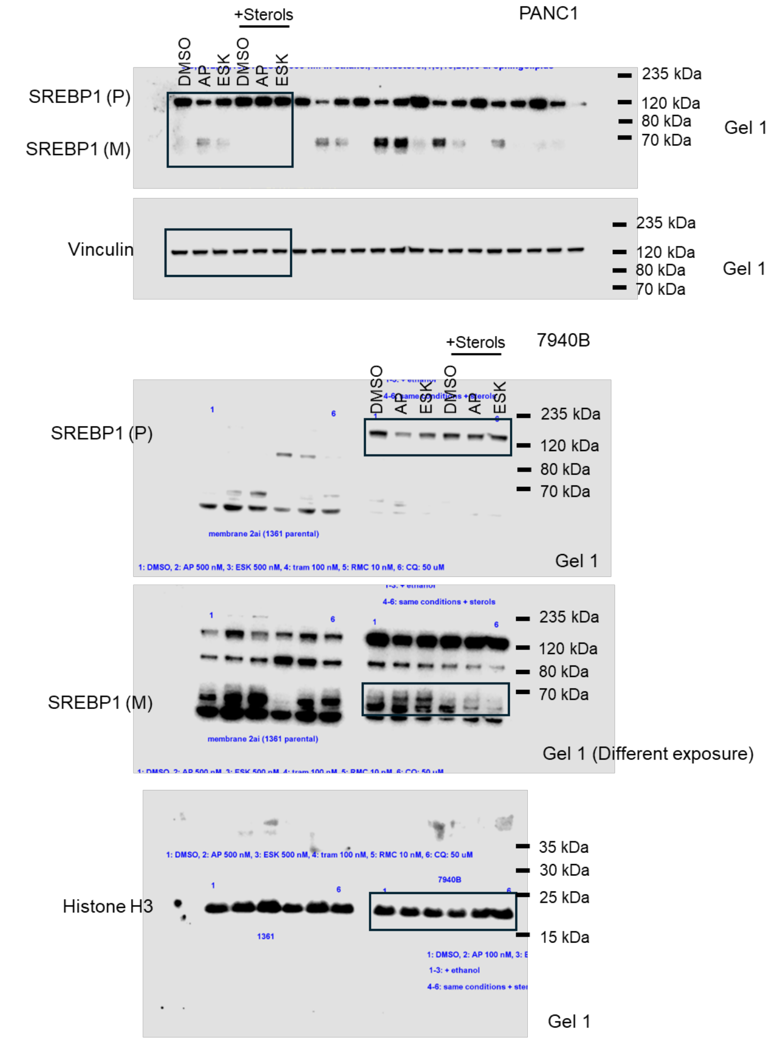

Extended Data Fig. 11b- MIA PaCa-2 and 7940B

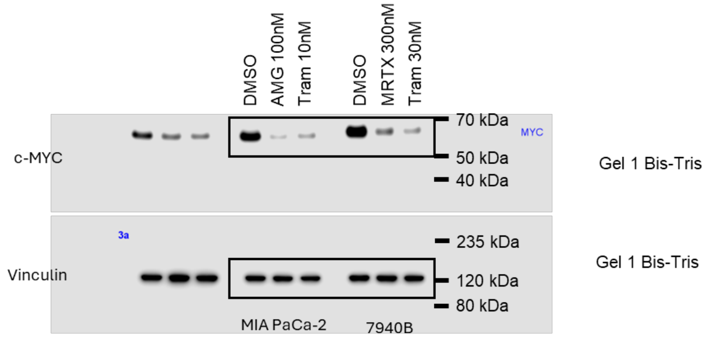

Extended Data Fig. 11 g- iKras 9805

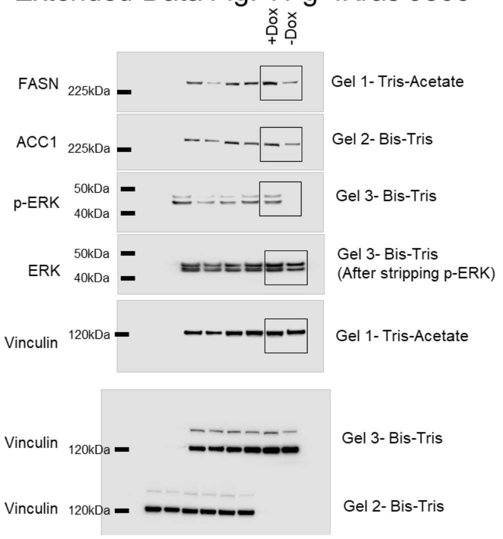

Scheme 1. Synthesis of Compound **PIK5-33d** [a]

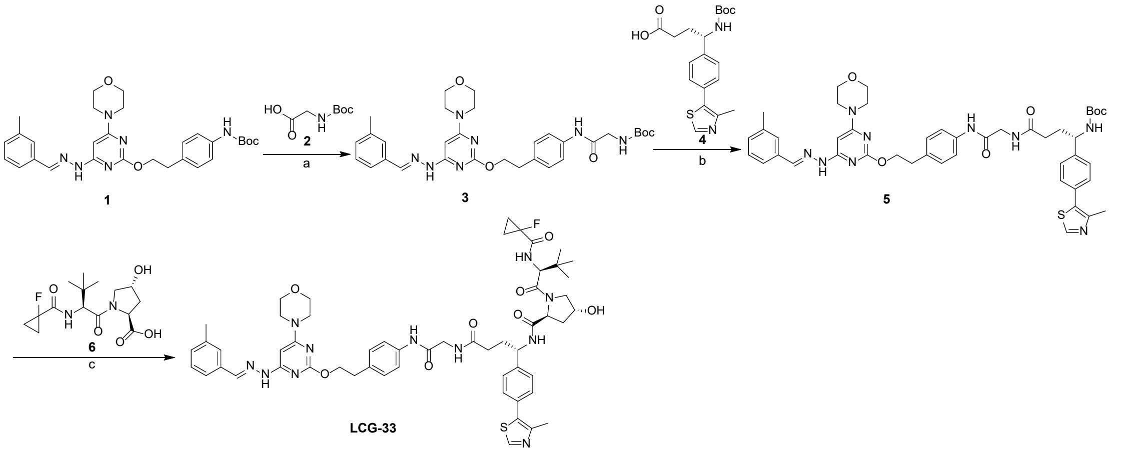

[a] **Reagents and conditions**: (a) i) trifluoroacetic acid (TFA), dichloromethane (CH_2Cl_2), room temperature (RT), 2 h; ii) 2-(7-azabenzotriazol-1-yl)-*N′*, *N′*, *N′*-tetramethyluronium hexafluorophosphate (HATU), triethylamine (Et_3N), *N*, *N*-dimethylformamide (DMF), RT, 4 h, 66% over two steps; (b) i) TFA, CH_2Cl_2, RT, 2 h; ii) HATU, Et_3N, DMF, RT, 4 h, 56% over two steps; (c) i) TFA, CH_2Cl_2, RT, 2 h; ii) HATU, Et_3N, DMF, RT, 4 h, 61% over two steps.

Chemistry Methods

tert-Butyl (*E*)-(2-((4-(2-((4-(2-(3-methylbenzylidene)hydrazineyl)-6-morpholinopyrimidin-2-yl)oxy)ethyl)phenyl)amino)-2-oxoethyl)carbamate (**3**)

The preparation of compound **1** has been reported in our previous study (ref: https://pubs.acs.org/doi/10.1021/acs.jmedchem.3c00912). To a solution of compound **1** (300 mg, 0.6 mmol, 1.0 equiv) in CH_2Cl_2 (10 mL) was added TFA (3 mL). The reaction mixture was stirred at RT for 2 h before being concentrated. The resulting residue was redissolved in DMF (10 mL). Further (*tert*-butoxycarbonyl)glycine (118 mg, 0.7 mmol, 1.2 equiv), Et_3N (285 mg, 360 μL, 2.8 mmol, 5.0 equiv) and HATU (319 mg, 0.8 mmol, 1.5 equiv) was then added. The resulting solution was stirred at RT for another 4 h, then diluted with H_2O (40 mL) and extracted with ethyl acetate (EtOAc, 30 mL). The aqueous phase was separated. The organic phase was washed with H_2O, dried with anhydrous sodium sulfate (Na_2SO_4), filtered and concentrated. The resulting residue was purified by column chromatography (MeOH/CH_2Cl_2) to obtain intermediate **3** (219

mg, 66%) as a white solid. ^{1}H NMR (600 MHz, DMSO-d_6) δ 10.86 (s, 1H), 9.86 (s, 1H), 7.99 (s, 1H), 7.50 (dd, *J* = 8.3, 6.8 Hz, 3H), 7.48 (s, 1H), 7.29 (t, *J* = 7.6 Hz, 1H), 7.23 (d, *J* = 8.5 Hz, 2H), 7.17 (d, *J* = 7.5 Hz, 1H), 7.02 (t, *J* = 6.0 Hz, 1H), 6.06 (s, 1H), 4.34 (t, *J* = 7.1 Hz, 2H), 3.71 (d, *J* = 5.8 Hz, 2H), 3.68 – 3.64 (m, 4H), 3.55 – 3.49 (m, 4H), 2.93 (t, *J* = 7.0 Hz, 2H), 2.34 (s, 3H), 1.40 (s, 9H). ^{13}C NMR (150 MHz, DMSO-d_6) δ 167.93, 164.27, 163.85, 163.41, 163.30, 155.82, 141.09, 137.82, 137.10, 137.00, 134.66, 133.09, 129.65, 129.07, 128.50, 126.91, 123.44, 119.02, 118.93, 77.92, 75.55, 66.40, 65.80, 44.11, 43.61, 34.15, 28.10, 20.83. LC−MS: calcd for $C_{31}H_{39}N_7O_5$ [M + H]$^+$ 589.3, found 589.4.

tert-Butyl (*S*,*E*)-(4-((2-((4-(2-((4-(2-(3-methylbenzylidene)hydrazineyl)-6-morpholinopyrimidin-2-yl)oxy)ethyl)phenyl)amino)-2-oxoethyl)amino)-1-(4-(4-methylthiazol-5-yl)phenyl)-4-oxobutyl)carbamate (**5**)

Intermediates **5** was prepared via an analogous procedure of intermediate **3**. ^{1}H NMR (600 MHz, DMSO-d_6) δ 10.87 (s, 1H), 9.89 (s, 1H), 8.98 (s, 1H), 8.14 (t, *J* = 5.8 Hz, 1H), 7.99 (s, 1H), 7.54 – 7.44 (m, 7H), 7.40 (d, *J* = 8.1 Hz, 2H), 7.29 (t, *J* = 7.6 Hz, 1H), 7.22 (d, *J* = 8.5 Hz, 2H), 7.17 (d, *J* = 7.5 Hz, 1H), 6.06 (s, 1H), 4.53 (dd, *J* = 15.0, 8.0 Hz, 1H), 4.34 (t, *J* = 7.0 Hz, 2H), 3.92 – 3.77 (m, 2H), 3.71 – 3.61 (m, 4H), 3.56 – 3.49 (m, 4H), 2.93 (t, *J* = 7.0 Hz, 2H), 2.47 (s, 3H), 2.34 (s, 3H), 2.24 – 2.13 (m, 2H), 1.92 – 1.83 (m, 2H), 1.38 (s, 9H). ^{13}C NMR (150 MHz, DMSO-d_6) δ 172.03, 167.60, 164.24, 163.82, 163.37, 155.01, 151.36, 147.68, 143.89, 141.14, 137.82, 137.03, 134.64, 133.17, 131.03, 129.71, 129.66, 129.06, 128.70, 128.50, 126.92, 126.79, 123.45, 119.11, 77.70, 75.54, 66.41, 65.80, 53.58, 44.11, 42.52, 34.15, 32.12, 28.17, 20.83, 15.92. LC−MS: calcd for $C_{45}H_{53}N_9O_6S$ [M + H]$^+$ 848.4, found 848.3.

(2*S*,4*R*)-1-((*S*)-2-(1-fluorocyclopropane-1-carboxamido)-3,3-dimethylbutanoyl)-4-hydroxy-*N*-((*S*)-4-((2-((4-(2-((4-(2-((*E*)-3-methylbenzylidene)hydrazineyl)-6-morpholinopyrimidin-2-yl)oxy)ethyl)phenyl)amino)-2-oxoethyl)amino)-1-(4-(4-methylthiazol-5-yl)phenyl)-4-oxobutyl)pyrrolidine-2-carboxamide (**PIK5-33d**)

PIK5-33d was prepared via an analogous procedure of intermediate **3**. ^{1}H NMR (600

MHz, DMSO-d_6) δ 10.86 (s, 1H), 9.89 (s, 1H), 8.99 (s, 1H), 8.58 (d, J = 8.3 Hz, 1H), 8.03 (t, J = 5.8 Hz, 1H), 7.99 (s, 1H), 7.54 – 7.48 (m, 3H), 7.48 – 7.44 (m, 3H), 7.39 (d, J = 8.3 Hz, 2H), 7.35 (dd, J = 9.4, 2.5 Hz, 1H), 7.29 (t, J = 7.6 Hz, 1H), 7.22 (d, J = 8.5 Hz, 2H), 7.17 (d, J = 7.5 Hz, 1H), 6.06 (s, 1H), 5.16 (d, J = 3.6 Hz, 1H), 4.84 (dd, J = 15.2, 7.7 Hz, 1H), 4.61 (d, J = 9.3 Hz, 1H), 4.50 (t, J = 8.4 Hz, 1H), 4.34 (t, J = 7.1 Hz, 2H), 4.29 (s, 1H), 3.86 (d, J = 5.8 Hz, 2H), 3.71 – 3.64 (m, 4H), 3.64 – 3.56 (m, 2H), 3.56 – 3.49 (m, 4H), 2.93 (t, J = 7.0 Hz, 2H), 2.47 (s, 3H), 2.37 – 2.31 (m, 4H), 2.28 – 2.22 (m, 1H), 2.10 – 2.04 (m, 1H), 2.00 – 1.92 (m, 2H), 1.79 – 1.72 (m, 1H), 1.41 – 1.31 (m, 2H), 1.26 – 1.18 (m, 2H), 0.97 (s, 9H). ^{13}C NMR (150 MHz, DMSO-d_6) δ 171.72, 170.20, 168.28, 167.55, 167.41, 167.00, 163.76, 163.34, 162.90, 150.90, 147.20, 142.66, 140.59, 137.31, 136.51, 134.15, 132.67, 130.47, 129.29, 129.14, 128.54, 128.25, 127.99, 126.40, 126.22, 122.93, 118.60, 78.18, 76.64, 75.05, 68.18, 65.88, 65.29, 58.21, 56.12, 55.99, 51.26, 43.60, 42.12, 37.16, 35.43, 33.64, 31.88, 31.55, 25.64, 20.32, 15.42, 12.41, 12.34, 12.12, 12.06. HRMS (ESI) calcd for $C_{55}H_{66}FN_{11}O_8S$ [M + H]$^+$ 1060.4879, found 1060.4879. HPLC purity 97.66%.

^{1}H-NMR spectra of compound PIK5-33d

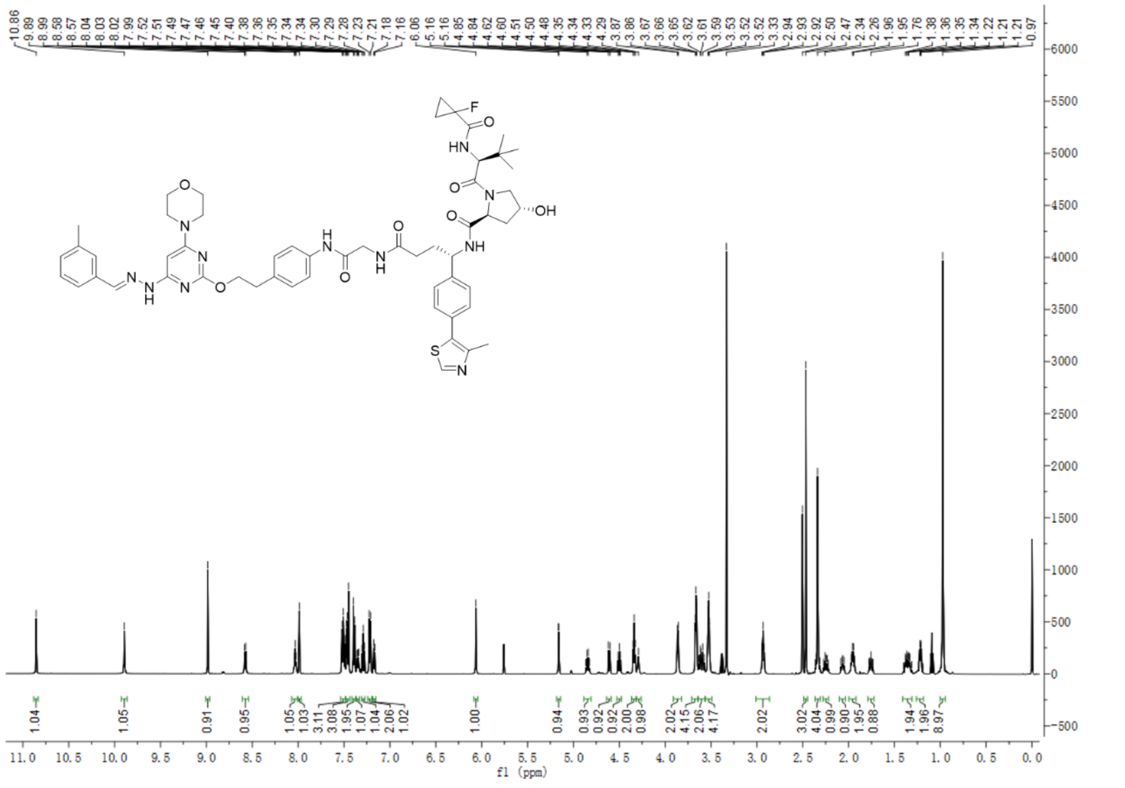

^{13}C-NMR spectra of compound PIK5-33d

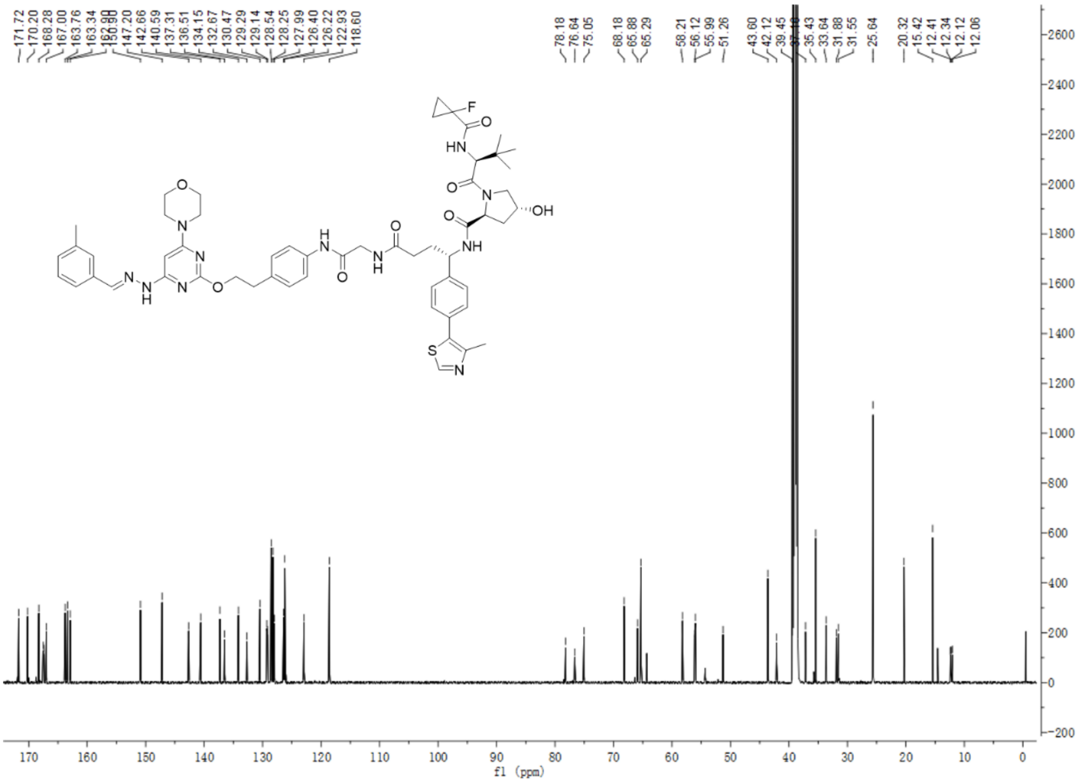

HPLC trace of compound PIK5-33d

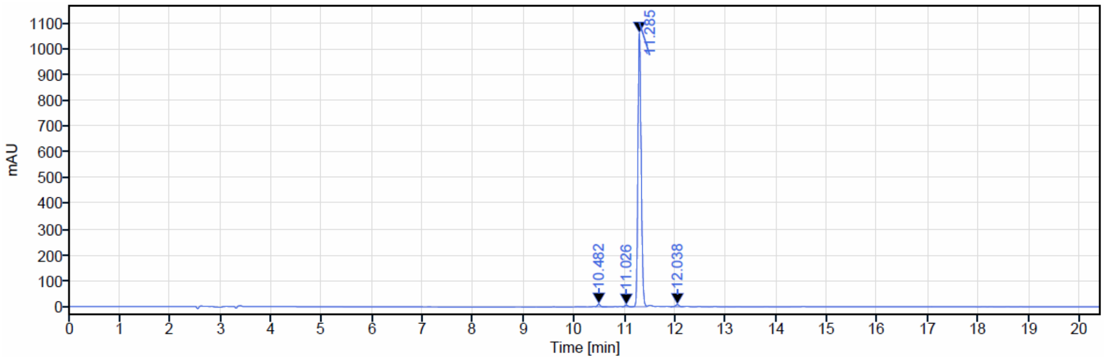

Signal: DAD1A,Sig=254,4 Ref=off

RT [min]	Type	Width [min]	Area	Height	Area%	Name
10.482	VB	0.23	44.10	11.03	0.90	
11.026	VB	0.18	28.45	6.95	0.58	
11.285	BV	0.31	4788.37	1066.25	97.66	
12.038	BV	0.25	42.38	8.44	0.86	
		**Sum**	4903.30		100.00	
