# Supplementary material for: Targeting PIKfyve-driven lipid metabolism in pancreatic cancer
Source: Nature. 2025 Apr 23;642(8068):776–84. doi: 10.1038/s41586-025-08917-z (PMC12176661; doi:10.1038/s41586-025-08917-z)
Supplement: Supplementary file 2 — Reporting Summary [file 41586_2025_8917_MOESM2_ESM.pdf]

nature portfolio

Corresponding author(s): Arul M. Chinnaiyan

Last updated by author(s): Feb 24, 2025

# Reporting Summary

Nature Portfolio wishes to improve the reproducibility of the work that we publish. This form provides structure for consistency and transparency in reporting. For further information on Nature Portfolio policies, see our Editorial Policies and the Editorial Policy Checklist.

## Statistics

For all statistical analyses, confirm that the following items are present in the figure legend, table legend, main text, or Methods section.

| n/a | Confirmed | |
|---|---|---|
| ☐ | ☒ | The exact sample size (*n*) for each experimental group/condition, given as a discrete number and unit of measurement |
| ☐ | ☒ | A statement on whether measurements were taken from distinct samples or whether the same sample was measured repeatedly |
| ☐ | ☒ | The statistical test(s) used AND whether they are one- or two-sided<br>*Only common tests should be described solely by name; describe more complex techniques in the Methods section.* |
| ☐ | ☒ | A description of all covariates tested |
| ☐ | ☒ | A description of any assumptions or corrections, such as tests of normality and adjustment for multiple comparisons |
| ☐ | ☒ | A full description of the statistical parameters including central tendency (e.g. means) or other basic estimates (e.g. regression coefficient) AND variation (e.g. standard deviation) or associated estimates of uncertainty (e.g. confidence intervals) |
| ☐ | ☒ | For null hypothesis testing, the test statistic (e.g. *F*, *t*, *r*) with confidence intervals, effect sizes, degrees of freedom and *P* value noted<br>*Give P values as exact values whenever suitable.* |
| ☒ | ☐ | For Bayesian analysis, information on the choice of priors and Markov chain Monte Carlo settings |
| ☒ | ☐ | For hierarchical and complex designs, identification of the appropriate level for tests and full reporting of outcomes |
| ☒ | ☐ | Estimates of effect sizes (e.g. Cohen's *d*, Pearson's *r*), indicating how they were calculated |

*Our web collection on statistics for biologists contains articles on many of the points above.*

## Software and code

Policy information about availability of computer code

| | |
|---|---|
| Data collection | No software was used for data collection. |
| Data analysis | Computational tools used:<br>Graphpad Prism 10 and included statistical tools<br>Fiji (ImageJ) (version 1.54i)<br>Imagestudio (version 5.2)<br>Tecan i-control (version 2)<br>SynergyFinder+ Web application 3.14.0<br>Incucyte 2023A Rev2 (2023.3.3.0 Rev2)<br>Seahorse Wave Controller Software (version2.6.3.5)<br>R (version 4.3.2)<br>cutadapt (version 4.1)<br>bowtie2 (version 2.4.5)<br>MAGeCK (version 0.5.9.5)<br>ggplot2 (version 3.4.4)<br>Quantstudio Design&Analysis Software (version 1.5.2)<br>bcl2fastq (version 2.20)<br>kallisto (version 0.50.1)<br>limma (version 3.58.1)<br>edgeR (version 4.0.16)<br>fgsea (version 1.28.0) |

Masshunter Workstation Quantitative Analysis for QQQ (version 10.1 Build 10.1.733.0)
Morpheus Matrix Visualization and analysis tool web application (no version information found)
Masshunter Quantitative Analysis (version 12.0)
Mass Profiler Professional (version 15.0)
lme4 (version 1.1-35.1)
Trimmomatic 0.39
Picard MarkDuplicates 2.26.0
samtools 1.9
bedtools 2.27.1
MACS2 2.2.7
wigToBigWig 2.4
FIMO 5.5.6
HOMER 5.1
BWA MEM 0.7.17

For manuscripts utilizing custom algorithms or software that are central to the research but not yet described in published literature, software must be made available to editors and reviewers. We strongly encourage code deposition in a community repository (e.g. GitHub). See the Nature Portfolio guidelines for submitting code & software for further information.

## Data

Policy information about availability of data

All manuscripts must include a data availability statement. This statement should provide the following information, where applicable:

- Accession codes, unique identifiers, or web links for publicly available datasets
- A description of any restrictions on data availability
- For clinical datasets or third party data, please ensure that the statement adheres to our policy

All source data and raw gel images are included with the manuscript. All materials are available from the authors upon reasonable request. All raw next-generation sequencing, such as DNA sequencing for the CRISPR screen and ChIP-seq or RNA-seq, have been deposited in the Gene Expression Omnibus (GEO) repository at NCBI with the accession numbers GSE255378 and GSE277832. Processed sequencing data, such as sgRNA counts and RNA-seq CPM, are included as Supplementary Table 1 and 3, respectively. Raw data for metabolomics and lipidomics experiments are included as Supplementary Tables (4 and 5, respectively). Other publicly available datasets were used: human genome assembly GRCh38: https://www.ncbi.nlm.nih.gov/datasets/genome/GCF_000001405.26/; murine genome assembly: GRCm38: https://www.ncbi.nlm.nih.gov/datasets/genome/GCF_000001635.20/, Hallin et al., 2019: GSE201412.

## Research involving human participants, their data, or biological material

Policy information about studies with human participants or human data. See also policy information about sex, gender (identity/presentation), and sexual orientation and race, ethnicity and racism.

| | |
|---|---|
| Reporting on sex and gender | Of the five patient samples from the University of Michigan pathological archives included in this study, four were from male patients and one was from a female patient. Gender was not reported. Sex and gender were not considered as variables in this study design. |
| Reporting on race, ethnicity, or other socially relevant groupings | All samples were from White, Non-Hispanic patients at the University of Michigan. |
| Population characteristics | There was no human population research in this study. |
| Recruitment | Patient tissues from biopsies of pancreatic tumors were acquired from the University of Michigan pathology archives. |
| Ethics oversight | Use of clinical formalin-fixed paraffin embedded specimens from the archives was approved by the University of Michigan Institutional Review Board and does not require patient consent. |

Note that full information on the approval of the study protocol must also be provided in the manuscript.

# Field-specific reporting

Please select the one below that is the best fit for your research. If you are not sure, read the appropriate sections before making your selection.

☒ Life sciences ☐ Behavioural & social sciences ☐ Ecological, evolutionary & environmental sciences

For a reference copy of the document with all sections, see nature.com/documents/nr-reporting-summary-flat.pdf

# Life sciences study design

All studies must disclose on these points even when the disclosure is negative.

| | |
|---|---|
| Sample size | Sample size was determined by preliminary studies and the level of the observed effect. |

| | |
|---|---|
| Data exclusions | Two datapoints were removed from lipidomics experiments as being statistical outliers. These are explicitly stated in the figure legends. No other data exclusions were made. |
| Replication | Independent experimental replicates for each experiment are explicitly stated in figure legends. In all instances, all attempts at replicating the experiments produced similar results. |
| Randomization | For animal studies, mice were randomly assigned to treatment groups. For all in vitro experiments, we used a common cell suspension to plate for both control and treatment groups. For retrospective pathological analyses or human samples, matched control and experimental sections were taken from all samples and thus did not require randomization. |
| Blinding | All histo-pathological evaluations of tissues and IHC/RNA-ISH-based scoring were carried out in a blinded manner by two independent pathologists. For animal studies, the researcher was blinded to group when measuring tumors. For all other experiments, the analyses did not require blinding as data quantification was carried out using instruments and automated workflows. |

# Reporting for specific materials, systems and methods

We require information from authors about some types of materials, experimental systems and methods used in many studies. Here, indicate whether each material, system or method listed is relevant to your study. If you are not sure if a list item applies to your research, read the appropriate section before selecting a response.

## Materials & experimental systems

| n/a | Involved in the study |
|---|---|
| ☐ | ☒ Antibodies |
| ☐ | ☒ Eukaryotic cell lines |
| ☒ | ☐ Palaeontology and archaeology |
| ☐ | ☒ Animals and other organisms |
| ☒ | ☐ Clinical data |
| ☒ | ☐ Dual use research of concern |
| ☒ | ☐ Plants |

## Methods

| n/a | Involved in the study |
|---|---|
| ☐ | ☒ ChIP-seq |
| ☒ | ☐ Flow cytometry |
| ☒ | ☐ MRI-based neuroimaging |

## Antibodies

Antibodies used

Human PIKfyve Antibody R&D Systems AF7885
SQSTM1/p62 (D5L7G) Mouse mAb Cell Signaling Technology 88588S
Cleaved PARP (Asp214) (D64E10) XP® Rabbit mAb Cell Signaling Technology 5625S
SQSTM1/p62 (D6M5X) Rabbit mAb Cell Signaling Technology 23214s
LC3A/B (D3U4C) XP® Rabbit mAb Cell Signaling Technology 12741S
Vinculin (E1E9V) XP® Rabbit mAb (HRP Conjugate) Cell Signaling Technology 18799S
GAPDH (14C10) Rabbit mAb (HRP Conjugate) Cell Signaling Technology 3683S
Recombinant Anti-Fatty Acid Synthase antibody [EPR7466] Abcam ab128856
Phospho-Acetyl-CoA Carboxylase (Ser79) (D7D11) Rabbit mAb Cell Signaling Technology 11818S
Acetyl-CoA Carboxylase (C83B10) Rabbit mAb Cell Signaling Technology 3676S
Anti-SREBP1 antibody Abcam ab28481
Histone H3 (96C10) Mouse mAb Cell Signaling Technology 3638S
Phospho-p44/42 MAPK (Erk1/2) (Thr202/Tyr204) (D13.14.4E) XP® Rabbit mAb Cell Signaling Technology 4370S
p44/42 MAPK (Erk1/2) (137G5) Cell Signaling Technology 4695S
Amersham ECL Peroxidase (HRP) Anti-Mouse IgG F(ab')2 Fragment Sheep Secondary Antibody Fisher/GE Healthcare NA931
Sheep IgG Horseradish Peroxidase-conjugated Antibody R&D Systems HAF016
anti-Rabbit IgG, peroxidase-linked species-specific whole antibody (from donkey) Secondary Antibody, Cytiva GE Healthcare UK Limited/Fisher NA934
Anti-Cytokeratin 19 antibody [EP1580Y] Abcam ab52625
CONFIRM Anti-Ki67 antibody Ventana 790-4286
Anti-Insulin (C27C9) Rabbit mAb Cell Signaling Technology 3014S
Anti-c-Myc antibody [Y69] - ChIP Grade Abcam ab32072
Anti-SPTLC1 antibody Abcam ab176706
SPTLC2 Polyclonal Antibody Invitrogen PA5-21142
AMPKα Antibody #2532 Cell Signaling Technology 2532S
Atg5 (D5F5U) Rabbit mAb #12994 Cell Signaling Technology 12994S
Atg7 (D12B11) Rabbit mAb #8558 Cell Signaling Technology 8558S
Anti-LDL Receptor antibody [EP1553Y] (ab52818) Abcam ab52818
XP® Anti-LAMP1 Rabbit Monoclonal Antibody [D2D11] Cell Signaling Technology 9091S
Goat Anti-Rabbit IgG Antibody (Alexa Fluor® 594) Jackson ImmunoResearch 111-585-045

Validation

Human PIKfyve Antibody WB validated; human
SQSTM1/p62 (D5L7G) Mouse mAb KO validated; human, mouse, rat, monkey
Cleaved PARP (Asp214) (D64E10) XP® Rabbit mAb drug validated; human, mouse, monkey
SQSTM1/p62 (D6M5X) Rabbit mAb KO validated; mouse, rat
LC3A/B (D3U4C) XP® Rabbit mAb drug validated; human, mouse, rat

Vinculin (E1E9V) XP® Rabbit mAb (HRP Conjugate) WB validated; human, mouse, rat, monkey, dog
GAPDH (14C10) Rabbit mAb (HRP Conjugate) WB validated; human, mouse, rat, monkey, bovine, pig
Recombinant Anti-Fatty Acid Synthase antibody [EPR7466] KO validated; human, mouse, rat
Phospho-Acetyl-CoA Carboxylase (Ser79) (D7D11) Rabbit mAb drug validated; human, mouse, rat
Acetyl-CoA Carboxylase (C83B10) Rabbit mAb AB dilution validated; human, mouse, rat, hamster
Anti-SREBP1 antibody WB validated; human, mouse, rat
Histone H3 (96C10) Mouse mAb WB validated; human, mouse, rat, monkey, zebrafish, bovine, pig
Phospho-p44/42 MAPK (Erk1/2) (Thr202/Tyr204) (D13.14.4E) XP® Rabbit mAb drug validated; human, mouse, rat, hamster, monkey, mink, D. melanogaster, zebrafish, bovine, dog, pig, S. cerevisiae
p44/42 MAPK (Erk1/2) (137G5) siRNA validated; human, mouse, rat, hamster, monkey, mink, D. melanogaster, zebrafish, bovine, dog, pig, C. elegans
Anti-Cytokeratin 19 antibody [EP1580Y] WB validated; human, mouse
CONFIRM Anti-Ki67 antibody Validated for IHC
Anti-Insulin (C27C9) Rabbit mAb AB dilution validated; human, mouse, rat
Anti-c-Myc antibody [Y69] - ChIP Grade drug validated; human, mouse, rat
Anti-SPTLC1 antibody WB validated; human, mouse
SPTLC2 Polyclonal Antibody KD verified; human, mouse
AMPKα Antibody #2532 WB validated; human, mouse, rate, hamster, monkey
Atg5 (D5F5U) Rabbit mAb #12994 KO verified; human, mouse, rat
Atg7 (D12B11) Rabbit mAb #8558 siRNA validated; human, mouse, rat

# Eukaryotic cell lines

Policy information about cell lines and Sex and Gender in Research

| | |
|---|---|
| Cell line source(s) | PANC-1, MIA PaCa-2, Panc 04.03, SW1990, Panc 10.05, and HPAF-II were obtained from American Type Culture Collection. 7940B was generously provided by Greggory Beatty, M.D., Ph.D. at Perlman School of Medicine at the University of Pennsylvania. The iKRAS 9805 cell line was previously described and was provided by Dr. Marina Pasca di Magliano at the University of Michigan. The UM PDAC primary cell cultures lines (UM2, UM19) were obtained from surgically resected samples from female patients and established through murine xenograft. KPC-1344 and KPC-1361 cells were derived from female KPC mice in-house according to described methods. |
| Authentication | All human cell lines were authenticated with STR genetic testing |
| Mycoplasma contamination | All cell lines were biweekly tested to be free of mycoplasma contamination. |
| Commonly misidentified lines (See ICLAC register) | None |

# Animals and other research organisms

Policy information about studies involving animals; ARRIVE guidelines recommended for reporting animal research, and Sex and Gender in Research

| | |
|---|---|
| Laboratory animals | For xenograft studies, 6-8-week-old CB17 severe combined immunodeficiency (SCID) mice obtained from the University of Michigan breeding colony were used. For syngeneic studies, 6-8-week-old C57BL6 mice obtained from Jackson Laboratories were used. For the autochthonous model, KPC animals were studied beginning at 10 weeks of age. For the prophylactic study, KPC animals were studied beginning at 6 weeks of age. For the Pikfyve GEMM studies, animals were studied for survival throughout their lifespan. Cohorts were taken for histological analyses at the indicated timepoints in the manuscript. |
| Wild animals | None used. |
| Reporting on sex | Both male and female mice were used throughout the study. Differences in sex were not observed. |
| Field-collected samples | None. |
| Ethics oversight | Experiments involving the Pdx1-Cre animals for the autochthonous model efficacy study were approved by the University of Glasgow Animal Welfare and Ethical Review Board and were performed under a UK Home Office license. All other animals used in this study were housed at the University of Michigan in a pathogen-free environment, and all procedures involving these animals were performed in accordance with requirements of the University of Michigan Institutional Animal Care & Use Committee (IACUC). |

Note that full information on the approval of the study protocol must also be provided in the manuscript.

## Plants

Seed stocks

Not applicable

Novel plant genotypes

Not applicable

Authentication

Not applicable

## ChIP-seq

### Data deposition

☒ Confirm that both raw and final processed data have been deposited in a public database such as GEO.

☒ Confirm that you have deposited or provided access to graph files (e.g. BED files) for the called peaks.

Data access links
*May remain private before publication.*

https://www.ncbi.nlm.nih.gov/geo/query/acc.cgi?acc=GSE277832 token: uruxgeokppaxnwx

Files in database submission

mctp_SI_37818_H5F2HDRX5_1_1.fq.gz
mctp_SI_37818_H5F2HDRX5_1_2.fq.gz
mctp_SI_37818_H5F2HDRX5_2_1.fq.gz
mctp_SI_37818_H5F2HDRX5_2_2.fq.gz
mctp_SI_37819_H5F2HDRX5_1_1.fq.gz
mctp_SI_37819_H5F2HDRX5_1_2.fq.gz
mctp_SI_37819_H5F2HDRX5_2_1.fq.gz
mctp_SI_37819_H5F2HDRX5_2_2.fq.gz
mctp_SI_37820_H5F2HDRX5_1_1.fq.gz
mctp_SI_37820_H5F2HDRX5_1_2.fq.gz
mctp_SI_37820_H5F2HDRX5_2_1.fq.gz
mctp_SI_37820_H5F2HDRX5_2_2.fq.gz
mctp_SI_37821_H5F2HDRX5_1_1.fq.gz
mctp_SI_37821_H5F2HDRX5_1_2.fq.gz
mctp_SI_37821_H5F2HDRX5_2_1.fq.gz
mctp_SI_37821_H5F2HDRX5_2_2.fq.gz
miapaca2_dmso_igg_peaks.bed
miapaca2_dmso_myc_peaks.bed
miapaca2_amg510_myc_peaks.bed
miapaca2_tram_myc_peaks.bed
miapaca2_dmso_igg.bw
miapaca2_dmso_myc.bw
miapaca2_amg510_myc.bw
miapaca2_tram_myc.bw

Genome browser session
(e.g. UCSC)

N/A

### Methodology

Replicates

One sample per condition

Sequencing depth

reads are 151-base, paired end
miapaca2_dmso_igg: raw reads: 26596872, uniquely mapped reads: 18164562
miapaca2_dmso_myc: raw reads: 29052973, uniquely mapped reads: 18490903
miapaca2_amg510_myc: raw reads: 30297035, uniquely mapped reads: 18729003
miapaca2_tram_myc: raw reads: 27112748, uniquely mapped reads: 17822895

Antibodies

Anti-c-Myc antibody [Y69] - ChIP Grade (ab32072)

Peak calling parameters

Trimmed, paired reads were aligned to the human reference genome (hg38, obtained from UCSC) using bwa mem 0.7.17 with options -5 -S -P -T 0. Alignments were filtered for mapping quality >= 20 using samtools 1.9. Read duplicates were removed using Picard MarkDuplicates 2.26.0. Non-primary alignments were removed using samtools view (option -F 0x900) and converted to BED format using bedtools bamtobed 2.27.1. Peaks were called from these alignments using MACS2 2.2.7.1 with default settings (peaks filtered at q < 0.05) and the -B option to generate bedGraph coverage files. Peaks were then filtered using the ENCODE Unified GRCh38 Exclusion List (https://www.encodeproject.org/files/ENCFF356LFX/). Coverages captured by MACS2 were converted to

bigWig using wigToBigWig 2.4.

Data quality

Quality was checked using the following parameters which yielded the following results:
miapaca2_dmso_igg: peaks (q < 0.05): 2966, peaks (q < 0.05 and enrichment > 5): 821
miapaca2_dmso_myc: peaks (q < 0.05): 9552, peaks (q < 0.05 and enrichment > 5): 3355
miapaca2_amg510_myc: peaks (q < 0.05): 10378, peaks (q < 0.05 and enrichment > 5): 2279
miapaca2_tram_myc: peaks (q < 0.05): 5431, peaks (q < 0.05 and enrichment > 5): 1691

Software

Basecalling: bcl2fastq 2 (Illumina)
Trimming: Trimmomatic 0.39
Alignment: BWA MEM 0.7.17
Alignment manipulation/filtering: samtools 1.9
Duplicate identification: Picard MarkDuplicates 2.26.0
BED file manipulation: Bedtools 2.27.1
Peak calling: MACS2 2.2.7.1
Coverage generation: MACS2 2.2.7.1 and wigToBigWig 2.4
Motif analysis: FIMO 5.5.6 and HOMER 5.
